# Supplementary material for: Coverage, delivery models, and implementation challenges of the community driven nutritional supplementation initiative for people with TB: A mixed methods study from Puducherry, India
Source: PLOS Glob Public Health. 2025 Dec 23;5(12):e0005477. doi: 10.1371/journal.pgph.0005477 (PMC12725536; doi:10.1371/journal.pgph.0005477)
Supplement: S1 Text — (DOCX) [file pgph.0005477.s001.docx]

**S1 Text: Operational Definitions**

1. **Presumptive pulmonary TB**: Refers to a person with any of the symptoms and signs suggestive of TB including cough more than 2 weeks, fever more than 2weeks, significant weight loss, haemoptysis, any abnormality in chest radiograph [1].
2. **Presumptive extrapulmonary TB:** Refers to the presence of organ specific symptoms and signs like swelling of lymph nodes, pain and swelling of joints, neck stiffness, disorientation, etc and /or constitutional symptoms like significant weight loss, persistent fever for more than 2 weeks [1].
3. **Presumptive drug resistant TB***:* those patients with TB who have failed treatment with first – line drugs, pediatric TB non-responders, patients with TB who are contacts of DR-TB, previously treated TB cases, patients with HIV and TB coinfection and patients with TB who are found positive on any follow up sputum smear examination during treatment with first – line drugs [1].
4. **Bacteriologically confirmed TB:** Is one from whom a biological specimen is positive by smear microscopy, culture/ WRD (such as xpert MTB/ RIF) [1].
5. **Clinically diagnosed TB:** presumptive TB who does not fulfil the criteria for bacteriological confirmation but has been diagnosed with active TB by a clinician or other medical practitioner who has decided to give the patient a full course of tb treatment [1].
6. **New patients:** Patient with TB who have never been treated for TB or has taken anti TB drugs for less than 1 month [1].

**Retreatment cases** include definitions from 7-9 [1].

1. **Recurrent TB**: Patients with TB who were previously diagnosed as successfully treated (cured/ treated completed) subsequently found to be microbiologically confirmed TB.
2. **Treatment after failure:** those who have been previously treated for TB and whose treatment failed at the end of their most recent course of treatment
3. **Treatment after loss of follow up:**  Patient with TB who was previously treated and was declared loss to follow up in their most recent course of treatment and subsequently found microbiologically confirmed TB.
4. **Household contact:** is a person who shared the same enclosed living space as the index TB patient for one/ more nights/ for frequent / extended daytime period during the 3 months before the start of current TB treatment [2].
5. **Fixed dose combination**: includes administration of isoniazid, rifampicin, pyrazinamide and Ethambutol as a daily dosage pill depending on the weight band for 2 months in intensive phase. This will be followed by HRE regime daily for 4 months in continuation phase [1].
6. **Organogram for National TB Elimination Programme in India** [3]

1. **Tuberculosis unit**: One TB unit caters to a population of 1.5-2.5 lakh population [1].
2. **Peripheral Health institution**: is a health facility manned by a Medical Officer. It includes Primary Health Centres, Community Health Centre, District Hospitals, Medical Colleges, Speciality hospitals and TB hospitals [4].
3. **Senior Treatment Supervisor** (STS): Key programme staff working under NTEP at TU level. They have to ensure that NTEP policies are followed and results of treatment and laboratory examinations are promptly entered in the portal [5].
4. **TB Health Visitor** (TBHV):

Responsible for decentralisation of directly observed treatment short course in patients with Tb. They also maintain the TB treatment cards of the patients and assist the STS and District/State TB officer in surveillance and reporting [5].

**Fig A: Visual diagram of mixed methods study**


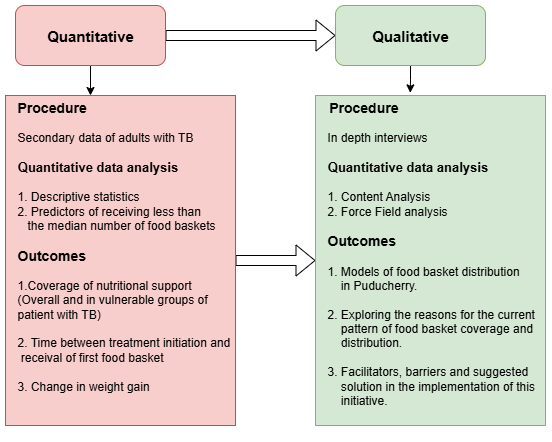


**Fig B: Various models of distribution of food baskets according to programmatic guidelines in India [6].**


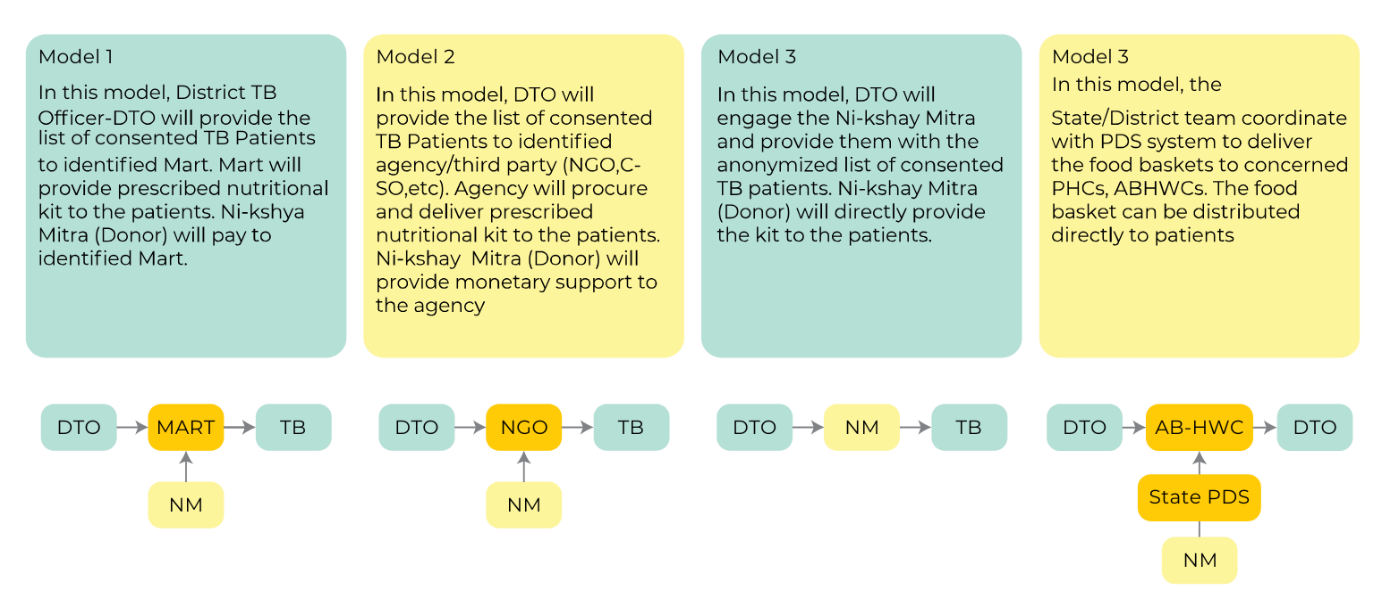


**References:**

1. Chaudhuri A. Recent changes in technical and operational guidelines for tuberculosis control programme in India - 2016: A paradigm shift in tuberculosis control. J Assoc Chest Physicians [Internet]. 2017 [cited 2025 Apr 9];5(1):1. Available from: https://journals.lww.com/ascp/fulltext/2017/05010/recent_changes_in_technical_and_operational.1.aspx

2. Ministry of Health and Family Welfare, Government of India. Guidelines for Programmatic Management of Tuberculosis Preventive Treatment in India [Internet]. Tbc Publications. 2021. p. 123. Available from: https://tbcindia.gov.in

3. Ministry of Health and Family Welfare CTD. INDIA TB REPORT 2024 NATIONAL TB ELIMINATION PROGRAMME [Internet]. New Delhi; 2025 [cited 2025 Apr 1]. Available from: http://www.tbcindia.gov.in

4. Health System Structure and functions for delivery of TB care [Internet]. Available from: https://tbcindia.gov.in/WriteReadData/l892s/3011812702TOG-Chapter 2-Health System Structure and functions for delivery of TB care.pdf

5. Ministry of Health and Family welfare, Government of India. Revised National Tuberculosis Control Program (RNTCP) Terms of Reference (TOR) for Appointment of Individual Consultants and Contractual Staff.

6. Ministry of Health and Family Welfare CTD. India TB Report 2023. 2023;1–105. Available from: https://tbcindia.gov.in/showfile.php?lid=3680
