# Supplementary material for: Coverage, delivery models, and implementation challenges of the community driven nutritional supplementation initiative for people with TB: A mixed methods study from Puducherry, India
Source: PLOS Glob Public Health. 2025 Dec 23;5(12):e0005477. doi: 10.1371/journal.pgph.0005477 (PMC12725536; doi:10.1371/journal.pgph.0005477)
Supplement: S2 Text — (DOCX) [file pgph.0005477.s002.docx]

**Additional File 2: Qualitative questionnaire and probes**

- **Interview guide to adults with TB who had not consented to the initiative:**

Following introduction of the participant and the interviewer

1. Were you aware of any monetary or nutritional support initiatives provided by the Government to the adults with TB?

1a. Tell us more about those schemes *(if `no` was the response to the above question otherwise skip to 2a/2b*)

1b. What were the sources from whom you have heard about this initiative?

1c. Could you tell us the reason for not enrolling in this initiative of receiving monthly nutritional support from the donors?

1d. In general, how did you fulfill the nutritional demands of yourself and your family in such a scenario?

**2a. For those with ongoing treatment:**

If a person was not aware of any initiative, then the interviewer will tell them about the nutritional supplementation initiative and link them to the respective TB program personnel for the same.

2b. **For those who had completed treatment:**

If a person was not aware, then the interviewer shall counsel them about the nutritional supplementation initiative for dissemination to the family members (identification of willing donors from TB affected households).

- **Interview guide to the adults with TB who had consented to this initiative:**

1. Were you aware of any monetary or nutritional support initiatives provided by the Government to the adults with TB?

1a. Tell us more about those schemes

1b. What were the sources from whom you have heard about this initiative?

1. How do you receive nutritional support presently (if currently on treatment) or how did you receive nutritional support earlier (if had completed treatment)?

2a. Could you give more detail as in through your family member / directly you received it? Whether you received at your home or at the treatment facility? (*If they could not understand question 2*)

2b. What were the problems you faced while you received the food baskets from the nearest health facility / home?

1. How many days do you think the baskets provided to you generally lasted?

3a. Why for ……days only? Did /do you share the food baskets provided to you amongst the family members?

3b. In your opinion, how was the quality and quantity of the contents within the food basket?

3c. Overall, can you recall, how many baskets you might have received during the treatment period or received till date?

3d. Can you substantiate the reasons behind adequate or inadequate receipt of food basket?

1. What were the food products that you had received from the donor so far?

4a. What were the food preferences in your opinion can be added to the routinely provided food items in the basket?

**Interview guide to the TB Program personnel (TB Health visitor)**

1. What were the challenges you generally faced while obtaining consent from adults with TB while enrolling in this nutritional support initiative?

1a. How did you try to resolve those issues?

1b. Reasons for the them declining support from your opinion

1. 2a. Have you approached the community / individuals for acting as donor in this initiative? **If yes then 2b to 2d**

2b. How do you approach the community / individuals for acting as donor in this initiative?

2c. Could you give an example for any such experience which led to identification of donor?

2d. What were the expectations from the donor in order to consent for this initiative?

1. How many health facilities were provided to you?

3a. How did you coordinate the nutritional kits received from the donor to the beneficiary enrolled in the facility from your areas?

3b. Was there any difference in the pattern of distribution (w.r.t quantity) between the health facilities you are providing support to? (may not be suitable for all)

3c. How did you ensure the quality or the quantity of the contents within the food basket?

1. What were the problems you faced while providing nutritional support to the adults with TB at those health facilities?

4a. From the donor’s side?

4b. From the adults with TB?

4c. How did you cope up with those problems?

4d. Any suggestive measures?

**Based on findings:**

4e. what did you do when there were limited supplies for a particular month from the donors? (do you prioritize?)

*Females*

More than 47 years

Rural (only in phi at rural areas)

PMDT and recurrent (low)

Extra pulmonary cases (low)

Diabetic population have received less than 3 baskets?

**Good practices:** Prioritizing less BMI, below poverty line and not working population

1. Any issues that you would like to share while reporting this activity of distribution of food baskets? (probe*: Ni-Kshay Mitra module within the Nikshay portal)*
2. *What were the challenges in obtaining repeated weight measurement in adults with TB during the follow up treatment period?*

- **Interview guide to the TB Program personnel (Senior TB treatment Supervisor)**

1. How did you approach the community / individuals for acting as donor in this initiative?

1a. Could you give an example for any such experience which led to identification of donor?

1b. Can you comment on the *Ni-Kshay* *Mitra* distribution pattern?

1. How many health facilities and TBHVs were provided to you for supervision?

2a. How did you manage supervising the nutritional kits distribution from different health facility in your area?

2b. Any best practices/methods you had experienced in sustaining the support of supply from donors and ensure smooth coordination?

2c. How did you ensure the quality or the quantity of the contents within the food basket?

2d. What were the expectations from the donor in order to consent for this initiative?

1. What were the difficulties in areas with no TBHV that you encounter while distributing food baskets?
2. What were the problems you faced while supervising the delivery of nutritional support to the adults with TB at any of those / all health facilities?

3a. From the donor’s side?

3b. From the adults with TB?

3c. How did you handle with those problems?

3d. Any suggestive measures?

3e. what did you do when there were limited supplies for a particular month from the donors?

1. **Comment on the**

- FB distribution
- Those PHI with less than 50% receival of FB among all the notified adults with TB
- Time to initiation of beyond 40 days?
- **Interview guide to the Medical Officers**

1. What was your role in approaching the community / individuals for acting as donor in this initiative?

1a. Could you give an example for any such experience which led to identification of donor?

1. Any best practice /methods you had experienced in sustaining the support of supply from donors and ensure smooth coordination?

2a. How did you ensure the quality or the quantity of the contents within the food basket?

1. What were the problems you faced while supervising the delivery of nutritional support to the adults with TB at your health facility?

3a. From the donor’s side?

3b. From the adults with TB?

3c. How did you handle with those problems?

3d. Any suggestive measures?

3e. what did you do when there were limited supplies for a particular month from the donors?

1. What were the food preferences in your opinion can be added to the routinely provided food items in the basket?
2. What IEC measures are undertaken to sensitize the public about this initiative?

**Interview guide to the *Ni-Kshay Mitra’s*:**

1. Tell us more about the *Ni-Kshay Mitra* or nutrition supplementation initiative you had enrolled under?

1a. What were the sources from whom you have heard about this initiative?

1b. Why did you enroll in this initiative? (any reasons)

1c. Share your experience as a donor of nutritional supplements under this initiative?

1d. Financial management.

1. Were you aware or did you happen to meet the adults with TB those were linked to during the period of your nutritional support?

2a. How did you procure and schedule your delivery?

2b. What were/are the factors that motivated you to establish and sustain nutritional support throughout the period?

2c. How did you ensure the quality or the quantity of the contents within the food basket that was packed and distributed?

1. What were the problems you faced while distributing the food baskets?

3a. While coordinating with the health staffs and adults with TB?

3b. How did you handle with those problems?

3c. Any suggestive measures?

1. What were the benefits for you / your organization in enrolling in this initiative?

4a. Were you acclaimed for this initiative from anybody.

1. What were the contents within the food basket which you had distributed?
2. Will you recommend others to enroll in this scheme from your circle?

(Yes or no then why? already have then how many?)

1. Is there anything you observed that you would like to share for the betterment of this initiative?
