# Supplementary material for: Coverage, delivery models, and implementation challenges of the community driven nutritional supplementation initiative for people with TB: A mixed methods study from Puducherry, India
Source: PLOS Glob Public Health. 2025 Dec 23;5(12):e0005477. doi: 10.1371/journal.pgph.0005477 (PMC12725536; doi:10.1371/journal.pgph.0005477)
Supplement: S3 Text — (DOCX) [file pgph.0005477.s003.docx]

**S3 Text: Results of negative binomial mixed model regression**

**Table A: Socio demographic and clinical characteristics of adults with TB by the number of food basket distribution between April 2023 and October2024, Puducherry district, India (N= 1055)**

| **Characteristic** | | | | N | Total  N = 1055*^1^* | **>2 food baskets**  N = 517*^1^* | **<= 2 food baskets**  N = 538*^1^* | **Unadj**  **RR†** | | **95%CI** | **P value** | | |  |
| --- | --- | --- | --- | --- | --- | --- | --- | --- | --- | --- | --- | --- | --- | --- |
| **Individual characteristics** | | | |  |  |  |  |  | |  |  | | |  |
| Age (years) | | | | 1,055 |  |  |  |  | |  |  | | |  |
| Less than or equal to 47 years | | | |  | 542(51.4) | 271(52.4) | 271(50.4) | - | | - |  | | |  |
| More than 47 years | | | |  | 513(48.6) | 246(47.6) | 267(49.6) | 1.07 | | 0.90,1.28 | 0.4 | | |  |
| **Gender** | | | | 1055 |  |  |  |  | |  |  | | |  |
| Female | | | |  | 380(36) | 201(38.9) | 179(33.3) | - | | - |  | | |  |
| Male | | | |  | 671(63.6) | 313(60.5) | 358(66.5) | 1.11 | | 0.93,1.33 | 0.2 | | |  |
| Transgender | | | |  | 4(0.4) | 3(0.6) | 1(0.2) | 0.65 | | 0.13,3.27 | 0.6 | | |  |
| Residence | | | | 920 |  |  |  |  | |  |  | | |  |
| Rural | | | |  | 323(35.1) | 164(35.3) | 159(34.9) | - | | - |  | | |  |
| Urban | | | |  | 597(64.9) | 300(64.7) | 297(65.1) | 0.90 | | 0.65,1.23 | 0.5 | | |  |
| Occupation | | | | 674 |  |  |  |  | |  |  | | |  |
| Working | | | |  | 481(71.4) | 242(70.1) | 239(72.6) | - | | - |  | | |  |
| Not working | | | |  | 193(28.6) | 103(29.9) | 90(27.4) | 0.95 | | 0.74,1.23 | 0.7 | | |  |
| **Family level characteristics** | | | |  |  |  |  |  | |  |  | | |  |
| Socio Economic status | | | | 860 |  |  |  |  | |  |  | | |  |
| Above poverty line | | | |  | 121(14.1) | 57(12.4) | 64(16) | - | | - |  | | |  |
| Below poverty line | | | |  | 739(85.9) | 402(87.6) | 337(84) | 0.89 | | 0.68,1.18 | 0.4 | | |  |
| No of Household contacts in  median (IQR) | | | | 1,029 | 3(1,3) | 3(1,3) | 2.00(1,3) | 1 | | 0.93,1.17 | 0.4 | | |  |
| **Behavioural characteristics** | | | |  |  |  |  |  | |  |  | | |  |
| History of Tobacco use | | | | 1,055 |  |  |  |  | |  |  | | |  |
| No | | | |  | 893(84.6) | 429(83) | 464(86.2) | - | | - |  | | |  |
| Yes | | | |  | 135(12.8) | 76(14.7) | 59(11) | 0.99 | | 0.75,1.31 | >0.9 | | |  |
| Unknown | | | |  | 27.(2.6) | 12(2.3) | 15(2.8) | 1.06 | | 0.63,1.79 | 0.8 | | |  |
| History of alcohol use | | | | 1,055 |  |  |  |  | |  |  | | |  |
| No | | | |  | 828(78.5) | 396(76.6) | 432(80.3) | - | | - |  | | |  |
| Yes | | | |  | 192(18.2) | 107(20.7) | 85(15.8) | 1.04 | | 0.81,1.32 | 0.8 | | |  |
| Unknown | | | |  | 35(3.3) | 14(2.7) | 21(3.9) | 1.09 | | 0.70,1.69 | 0.7 | | |  |
| **BMI at baseline** | | | | 1,049 |  |  |  |  | |  |  | | |  |
| Beyond 18.5 | | | |  | 643(61.3) | 316(61.5) | 327(61.1) | - | | - |  | | |  |
| Undernutrition (16-18.4) | | | |  | 223(21.3) | 109(21.2) | 114(21.3) | 1.08 | | 0.87,1.33 | 0.5 | | |  |
| Severe undernutrition (<16) | | | |  | 183(17.4) | 89(17.3) | 94(17.6) | 1.07 | | 0.85,1.34 | 0.6 | | |  |
| **Diagnosis and Treatment details** | | | |  |  |  |  |  | |  |  | | |  |
| Site of Tuberculosis | | | | 1,054 |  |  |  |  | |  |  | | |  |
| Extra pulmonary | | | |  | 347(32.9) | 174(33.7) | 173(32.2) | - | | - |  | | |  |
| Pulmonary | | | |  | 703(66.7) | 339(65.7) | 364(67.7) | 1.05 | 0.88, 1.26 | | | 0.6 | |  |
| Both | | | |  | 4(0.4) | 3(0.6) | 1(0.2) | 0.95 | 0.25,3.62 | | | >0.9 | |  |
| Type of Tuberculosis | | | | 1,055 |  |  |  |  |  | | |  | |  |
| New | | | |  | 905(85.8) | 445(86.1) | 460(85.5) | - | - | | |  | |  |
| Drug resistant | | | |  | 18(1.7) | 7(1.4) | 11(2) | 1.23 | 0.70,2.18 | | | 0.5 | |  |
| Retreatment | | | |  | 132(12.5) | 65(12.6) | 67(12.5) | 0.99 | 0.82,1.37 | | | 0.7 | |  |
| Classification of Tuberculosis | | | | 1,012 |  |  |  |  |  | | |  | |  |
| Clinically diagnosed | | | |  | 289(28.6) | 142(28.6) | 147(28.5) | - | - | | |  | |  |
| Bacteriologically confirmed | | | |  | 723(71.4) | 354(71.4) | 369(71.5) | 1.01 | 0.83, 1.22 | | | >0.9 | |  |
| Drug combinations taken | | | | 1,050 |  |  |  |  |  | | |  | |  |
| Fixed dose combinations | | | |  | 981(93.4) | 489(94.8) | 492(92.1) | - | - | | |  | |  |
| Loose drugs | | | |  | 69(6.6) | 27(5.2) | 42(7.9) | 1.24 | 0.91,1.69 | | | 0.2 | |  |
| **Comorbidity status** | | | |  |  |  |  |  |  | | |  | |  |
| Diabetes Mellitus | | | | 1,036 |  |  |  |  |  | | |  | |  |
| No | | | | |  | 468(45.2) | 286(56.0) | 182(34.7) | - | - | | |  | |
| Yes | | | | |  | 389(37.5) | 183(35.8) | 206(39.2) | 1.28 | 0.94, 1.47 | | | 0.2 | |
| Unknown | | | | |  | 179(17.3) | 42(8.2) | 137(26.1) | 1.26 | 0.90, 1.75 | | | 0.2 | |
| HIV status | | | | | 1,042 |  |  |  |  |  | | |  | |
| Non-reactive | | | |  | 1,031(98.9) | 506(99) | 525(98.9) | - | - | | |  | |  |
| HIV reactive | | | |  | 3(0.3) | 0(0) | 3(0.6) | 1.86 | 0.69,4.99 | | | 0.2 | |  |
| Unknown | | | |  | 8(0.8) | 5(1) | 3(0.6) | 0.92 | 0.36,2.38 | | | 0.9 | |  |
|  | | |  |  | ^1n (%), † Unadj RR – unadjusted risk ratio^ | | | | | | | | | |
|  | | |  |  |  | | | | | | | | | |

The variables with P value less than 0.20 such as gender, drug combinations taken, those with diabetic status were considered. Also, variables from qualitative interviews that were the basis of prioritisation such as occupation, classification of tuberculosis, socio economic status and nutritional status (BMI) were applied for predicting those with a risk of getting less than or equal to two food baskets. None of the variables were found to be significant as shown in Supplementary Table 2.

**Table B: Socio demographic and clinical predictors of adults with TB receiving less than or equal to two food baskets between April 2023 and October2024, Puducherry district, India (N= 1055)**

| **Characteristic** | **Adj risk ratio** | **95% CI*^1^*** | **p-value** |
| --- | --- | --- | --- |
| **Gender** |  |  |  |
| Females | — | — |  |
| Males | 1.07 | 0.76, 1.51 | 0.7 |
| Transgender | 0.90 | 0.15, 5.49 | >0.9 |
| **Socio Economic status** |  |  |  |
| Above poverty line | — | — |  |
| Below poverty line | 0.83 | 0.57, 1.21 | 0.3 |
| **Occupation** |  |  |  |
| Working | — | — |  |
| Not working | 0.94 | 0.65, 1.36 | 0.8 |
| **BMI at baseline** |  |  |  |
| Beyond 18.5 | — | — |  |
| 16-18.4 | 0.99 | 0.71, 1.39 | >0.9 |
| <16 | 0.88 | 0.59, 1.31 | 0.5 |
| **Classification of Tuberculosis** |  |  |  |
| Clinically diagnosed | — | — |  |
| Bacteriologically confirmed | 1.02 | 0.75, 1.38 | >0.9 |
| **Drug combinations taken** |  |  |  |
| Fixed dose combinations | — | — |  |
| Loose drugs | 1.00 | 0.56, 1.79 | >0.9 |
| **Diabetes Mellitus** |  |  |  |
| No | — | — |  |
| Yes | 1.19 | 0.89, 1.60 | 0.2 |
| Unknown | 1.62 | 1.00, 2.62 | 0.05 |
| *^1^*CI = Confidence Interval | | | |
