# Supplementary material for: Coverage, delivery models, and implementation challenges of the community driven nutritional supplementation initiative for people with TB: A mixed methods study from Puducherry, India
Source: PLOS Glob Public Health. 2025 Dec 23;5(12):e0005477. doi: 10.1371/journal.pgph.0005477 (PMC12725536; doi:10.1371/journal.pgph.0005477)
Supplement: S1 GRAMMS Checklist — (DOCX) [file pgph.0005477.s005.docx]

# Good Reporting of a Mixed Methods Study (GRAMMS)

1. **Describe the justification for using a mixed methods approach to the research question**

This community-driven initiative was grounded in a real world setting with factors interplaying at the community (donor), within the health system at individual level. Hence, mixed method approach by pragmatic philosophy was suitable for deriving insights underlying the coverage and implementation. This has been provided in Page 21, line no 456-459.

1. **Describe the design in terms of the purpose, priority and sequence of methods**

This mixed methods study included secondary data in the quantitative phase followed by in-depth interviews in qualitative phase (sequential explanatory). This has been diagrammatically represented as a visual diagram (Fig A in S1 Text) consisting of the procedure, analysis and outcome of each method.

1. **Describe each method in terms of sampling, data collection and analysis**

- Details of our sampling, data collection and analysis were described in the methods section – Page 7-11

1. **Describe where integration has occurred, how it has occurred and who has participated in it**

- This was a sequential explanatory study design.
- The integration has been provided for each of the objective in **Fig 1** of the manuscript. This diagram also explains how the integration occurs.
- Participants in the qualitative phase has been detailed in Page 7-8 line no 143-158, verbatims of the results section and in **Table 2**.

1. **Describe any limitation of one method associated with the present of the other method**

Despite efforts to ensure data quality, some limitations of using secondary data may persist. However, efforts were made to triangulate the data of the nutritional support coverage from multiple data sources supported by qualitative interviews from different stakeholders. This has been mentioned in the strengths and limitations section in Page no: 22, Line no: 456-467.

1. **Describe any insights gained from mixing or integrating methods**
   - Gaps in prioritization attempts to ensure equitable distribution of food baskets to the vulnerable groups was triangulated using this integration methods.
   - Provided evidence for recommending context specific patient centric model. This was also mentioned in the recommendations. Page no:22, Line no:482-484
