# Supplementary material for: Coverage, delivery models, and implementation challenges of the community driven nutritional supplementation initiative for people with TB: A mixed methods study from Puducherry, India
Source: PLOS Glob Public Health. 2025 Dec 23;5(12):e0005477. doi: 10.1371/journal.pgph.0005477 (PMC12725536; doi:10.1371/journal.pgph.0005477)
Supplement: Attachment — Submitted filename: Response to reviewers.docx [file pgph.0005477.s006.docx]

| **Sl No** | **Reviewer 1 comments** | **Reply to the reviewer comments** | **Reference** |
| --- | --- | --- | --- |
| 1 | Title - accessibility as an outcome is not measured – has to be removed from the write up | Thank you for the comment. The title has been revised as suggested by the reviewer. | Page no 1, Line no: 3-4  Page no 3, Line no: 29-30 |
| 2 | Introduction – various outcomes of interest can be briefly justified | We acknowledge the comment. It has been provided as Supplementary Fig 1 in S1 File. | Supplementary Fig 1 in S1 File. |
| 3 | Introduction - novelty of the study remains questionable, | Thank you for the comment. But we have not claimed novelty of the study as OR/IR is context specific. Coverage and implementation challenges have never been assessed in Puducherry using mixed method approach, thus, compelling the need of conducting this study.  The findings from this study are relevant and have not been explored by any other studies, adding strength to the study.  Evidences suggest that currently there is no information about the total number of food baskets received by patient with TB unlike the total food baskets distributed annually in national reports. Refer to the reference number 13.  Also, there is dearth of data about the initiation of first food basket from the time of treatment initiation. This is of importance as the majority of TB deaths occur within first two months of treatment. These two points has been added in the introduction section as rationale. | Page no 5, Line no: 80-83 |
| 4 | Introduction - the expected outcome of improvement in nutrition among patient with TB can be included here. | It has been provided as Supplementary Fig 1 in S1 File and also in the methods section. | Supplementary Fig 1 in S1 File  Page no 6, Line no: 106-108 |
| 5 | Methods – using the Cohort study design is questionable – please include information on the two groups, follow up and assessment if retained as Cohort study | Thank you for the comment. The secondary data included information on a cohort of adults with TB notified at public facilities between 1st April 2023 and 31st March 2024. The exposure of interest was whether individuals had consented to or declined nutritional support. Their follow up outcomes included information about the number of food baskets received throughout their treatment period, treatment outcomes and changes in weight gain (retrospective cohort). | Page no 6, Line no: 104-108 |
| 6 | Methods -Elaborate on existing programme guidelines – about the collection and distribution of food baskets, for example various models of food basket delivery | Thank you for the comment. The various models of existing food basket distribution in India has been given provided in S1 File as supplementary Figure 2 with the addition of reference (13). | Supplementary Fig 2 in S1 File |
| 7 | Methods - It’s important to describe about Nikshay Poshan Yojana as it also addresses the nutritional needs of patients with TB | The following lines has been added in the introduction section, “National Tuberculosis Elimination Programme (NTEP) programme in India launched the Nikshay Poshan Yojana initiative in 2018 to support the nutrition needs of patients with TB (PwTB). Under this initiative, PwTB receive 1000 INR (~ 12 USD) per month through direct benefit transfer during their course of treatment. Also, the reference has been provided here as (12)”. | Page no 4, Line no: 66-69 |
| 8 | Methods-Mention the number of interviews conducted over phone | A total of 42 interviews were conducted, comprising of 33 face-to-face and 9 telephonic interviews with various stakeholders, including TB program personnel. This has been specified in response to the reviewer’s comments in the methods section. | Page no 8, Line no: 162-164 |
| 9 | Elaborate qualitative data analysis - how was transcription done, time lag between interviews and transcription | We appreciate this comment. All the audio-recorded interviews and the handwritten field notes were transcribed and translated from vernacular language to English manually within a week’s interval. | Page no 10, Line no: 218-220 |
| 10 | Operational definition – include definitions of coverage and delay, equity of distribution | Thank you for the comments. Has been mentioned in the data analysis section of quantitative phase where,  Coverage was defined as - number of PwTB wo have received at least one food basket among all those who have consented for the nutritional support.  The definition of delay was discussed with the NTEP Program manager of Puducherry where it is recommended to initiate nutritional support as early as possible following treatment initiation. However, there is currently no document to quote it as reference. Therefore, following discussion with all the co-authors, the term delay has been replaced as time interval from treatment initiation to the receipt of 1^st^ food basket throughout the manuscript.  Equity was assumed if the above two indicators were better among adults with very severe undernutrition and below poverty line. | Page no 10, Line no: 205-210 |
| 11 | Is it a mixed method study, does the? quantitative part answer any research questions like coverage or delay or just supports the qualitative data with description of available data from records. | It is a sequential explanatory mixed method study with collection of secondary data first followed by exploration of the findings in the qualitative phase. The reference of this has been quoted as (18).  The outcomes of quantitative phase have been provided in visual diagram (S1 File). This includes coverage, interval between treatment initiation and the receival of first food basket and change in weight gain.  The integration of the qualitative component as a continuation of quantitative findings has been provided in Fig 1. I hope this explanation helps. | Page no 5, Line no: 97-98. |
| 12 | Results - Figure 2 is not legible | The resolution of the Figure 2 has been enhanced and attached for reference. | Refer to Figure 2. |
| 13 | Results - Only 50 % received three food baskets – are the number of donors sufficient enough to provide food baskets to all the TB patients. The qualitative part should be strong to inform why only half of the eligibles received food baskets. | We acknowledge the comment. In has been supported as quotes in the results section. | Page no 13. Line no: 284-289 |
| 14 | Qualitative results can be presented in a better way – suggestion: the quotes supporting the codes and themes can be presented alongside in the table in a separate column. | Thank you for the comment. This comment has been discussed with all the co investigators. Although it was a sequential explanatory mixed methods study, we had a relatively small quantitative component and a large qualitative phase involving the stakeholders.  We feel that giving quote alongside the codes and themes may result in long table/ additional table. Therefore, it gets complex for the reader to refer each table for the same given theme.  We intend to produce context specific solutions for which the we assume that presentation of facilitators and barriers alongside would help. However, we have provided additional verbatims under appropriate sub heading. | - |
| 15 | Equity in distribution and monitoring of weight gain – these outcomes are abruptly coming into the write up in the results section | These outcomes been provided as Supplementary Fig 1 in S1 File and also in the methods section. | Supplementary Fig 1 in S1 File  Page no 10, Line no: 205-210 |
| 16 | Qualitative research was a good opportunity to explore challenges like – quality standards of the basket, whether the TB patients are actually consuming the food basket. If data is collected on the same, it can strengthen the discussion session | Thank you for the comment. Both of these comments were highlighted in Table 2. However, following discussion with all the co-investigators, we have added two sub sections on: “Ensuring the quality standards of the contents within the food basket” and “feedback about food baskets from the consented patients with TB”.  Within these sub sections, we have provided the supporting verbatims. | Page no 14 and 15. Line no: 309-323. |
|  | **Reviewer 2** |  |  |
| 1 | Title: Not clear and doesn’t reflect the full purpose of the study. Need to be edited. | Thank you for the comment. The old title has been replaced with the following title as, “Food baskets for people with TB in Puducherry, India: How many, how early and who received? | Page no 1, Line no: 3-4  Page no 3, Line no: 29-30 |
| 2 | Abstract: The method section of abstract is vague. Needs to be edited based on comments given for the method part below. | The abstract section has been modified based on the reviewer’s comments. | Page no 3, Line no: 35, 38-39 |
| 3 | Abstract Results should describe the specific themes of the qualitative study. | The abstract section has been modified based on the reviewer’s comments. | Page no 3, Line no: 43-45 |
| 4 | Abstract Conclusion must only reflect based on the findings presented in the paper. | The abstract section has been modified based on the reviewer’s comments. | Page no 3,4, Line no: 52-55 |
| 5 | Line 94-98: Study design is mentioned as “explanatory mixed methods operational research involved a cohort study (quantitative phase)” which doesn’t make anything clearer. Better to mention this a mixed method study (using secondary data for quantitative and IDI for qualitative) | Thank you for the comment. Has been subsequently modified in the study design section as: “This mixed methods study included secondary data in the quantitative phase followed by in-depth interviews in qualitative phase (sequential explanatory)”.  Mixed method studies are classified as sequential explanatory, exploratory, convergent parallel, embedded and multi-phase mixed study design. We have followed sequential explanatory study design. Hence, we have explained this in brief with references (18). | Page no 5, Line no:97-98  Page no 6, Line no 104-108 |
| 6 | Line 98: What is meant (by pragmatic philosophy)? Clarify this? Is this related to the theoretical framework for qualitative component? | Thank you for the comment. The pragmatic philosophy was employed in this study, as the implementation of this community-driven initiative was grounded in a real world setting with factors interplaying at the community (donor), within the health system and at individual levels. The reference number being (18). To clarify, this is not a theoretical framework.  Further, the insights from this pragmatic paradigm can guide the policy makers and program managers in making context-specific adaptations. | Page no 5-6, Line no:98-103. |
| 7 | Study population: The quantitative phase is based on secondary data and not based interviews with patients. Line 134-136 is giving a wrong perspective. | Thank you for the comment. Brief description has been provided. | Page no 7, Line no: 141-142 |
| 8 | Line 145_ What is meant by extreme or deviant sampling? Do authors mean positive deviance sampling? Why it was chosen? PD approach requires very different frame work and sequential steps and authors miss to mention any of these | Thank you for the comment. Extreme or deviant sampling is a sub type of purposive sampling.  The rationale of using extreme sampling in our study can be justified as we intended to generate insights from those PHIs with high and low coverage of food baskets distribution.  As it includes sample from both the spectrums, it does not fulfil the definition of positive deviance approach. Further explanation has been provided in the manuscript. | Page no 8, Line no: 154-157 |
| 9 | Data collection: Line 155-167: On what basis the time period of secondary data was decided ((1/4/2023-31/3/2024). Explain steps on how data quality was ensured? Missing data etc. Add a section quality control for both sets of data | Thank you for the comment. The time period from April 2023 was chosen as the initiative was launched nationally six months earlier in September 2022. We intended to follow up all individuals who consented to receive nutritional support till their treatment completion, which varied between six months to one year.  Information about missing data and ensuring data quality has been mentioned in the manuscript under the headings: data collection. | Page no 7. Line no: 142-145  Page no 9. Line no: 178-181  Page no 9-10. Line no: 195-197.  Page no 10. Line no: 218-220.  Page no 11. Line no: 231-234. |
| 10 | Overall provide COREQ checklist needs to provided for methodology (<https://onlinelibrary.wiley.com/pb-assets/assets/17416612/COREQ_Checklist-1556513515737.pdf>). | Thank you for the comment. As it is mixed methods study, we have used GRAMMS checklist (which is more suitable than COREQ) for reporting our study as per the reviewer comments. This has been mentioned in the manuscript and also provided as a supplementary file (S4 file). | Page no 11. Line no: 233-234. |
| 11 | Line: 197 Line Negative binomial mixed model regression to identify the predictors? Line 260: But why Poisson regression is mentioned in the result? Either way justify the use of the methods? Does outcome variable is skewed? | We acknowledge the comment. As the outcome variable (proportion who received less than the median number of food baskets) is skewed, we intended to use negative binomial mixed model regression. There was a typo error and kindly ignore the previous mention of Poisson regression. Necessary changes has been made to the manuscript file in the quantitative analysis section. | Page no 10. Line no: 212-213. |
| 12 | Qualitative analysis methods needs to spelled out clearly (COREQ needs to be followed). | As it is mixed method study, we have used GRAMMS checklist for reporting our study as per the reviewer comments. This has been mentioned in the manuscript and also provided as a supplementary file (S4 file). | Page no 11. Line no: 233. |
| 13 | Presenting only few broad themes is not appropriate for the qualitative analysis. Describe the framework uses, codebooks, sub themes etc in a flow chart. Describe saturation point. | Thank you for the comment. The use of framework, codebook and saturation point has been provided in qualitative data analysis section.  Further, codebook has also been added as supplementary file (S3 file).  Also, based on the comment of the reviewer, table 2 has been modified to include categories and sub categories. | Page number 11. Line no: 224-225  Page number 33-35, Table 2 |
| 14 | Result should provide perspectives of different stakeholders (MO, Frontline staff based on deviant category) which at present is missing | Thank you for the comment. Anonymous identifiers have been provided in Table 2 to outline the perspective of different stakeholder. | Page number 33-35, Table 2 |
| 15 | Findings of qualitative and quantitative data needs triangulation which at present is just spelled but not described in detail. | Thank you for the comment. In this study, we aimed at assessing the coverage and exploring the reasons for high and low coverage. Subsequently, we indented to identify the predictors of receiving less number of food baskets. Also, we were interested in studying the context specific model of food basket distribution. Apart from that, our intention was not to triangulate the findings.  For better clarity, we have added Supplementary Fig 1 in S1 File to distinguish the outcomes from both quantitative and qualitative phases.  Further, the integration of this has been provided in Fig 2. | Supplementary Fig 2 in S1 File |
| 16 | Recommendations section needs to be shortened. Don’t explain the follow up activities which is not related to the results. If so, then explain it as part of your study methodology under heading: participant engagement | Thank you for the comment.We have added a heading “Implications for policy and practice”. In this heading we have documented the changes in the systems that followed after the conduct of this OR study. | Page no 21, Line no 458-465 |
| 17 | Conclusion must only reflect the findings presented in the paper (Don’t mention the following Line 366-368: We observed a higher proportion of adults with TB to have consented and subsequently 367 received nutritional support compared to previous Indian study and can be directly attributed 368 to administrative commitment] | Thank you for the comment. The statement has been modified to reflect only the findings of the study. | Page no 18, Line no 402-403. |
| 18 | The manuscript needs complete English edition and need to ensure quality of formatting (especially the heading and sub heading needs to be done clearly which at present is very messy). Attach COREQ checklist mandatorily. | Thank you for the comment. The manuscript has been reviewed by two co-authors and edits has been made at appropriate places. Formatting of the headings is also complete as per the comments.  As it is mixed method study, we have used GRAMMS checklist for reporting our study as per the reviewer comments. | - |
