# Supplementary material for: Coverage, delivery models, and implementation challenges of the community driven nutritional supplementation initiative for people with TB: A mixed methods study from Puducherry, India
Source: PLOS Glob Public Health. 2025 Dec 23;5(12):e0005477. doi: 10.1371/journal.pgph.0005477 (PMC12725536; doi:10.1371/journal.pgph.0005477)
Supplement: Attachment — Submitted filename: Response_to_reviewers_auresp_2.docx [file pgph.0005477.s007.docx]

| **Sl No** | **Reviewer 1 comments** | **Reply to the reviewer comments** | **Reference** |
| --- | --- | --- | --- |
| **1** | Overall, the write-up has to be improved for English grammar | We have extensively worked on our manuscript’s language to improve the flow and readability. We have proof read it from English professional as well. And has been added in the acknowledgement section. | Page no 23. Line no: 522-524 |
| **2** | The entire study is largely descriptive and does not evaluate the programme. | Thank you for highlighting this important point.  The *Ni-Kshay Mitra* initiative was a part of the National TB elimination Programme in India that was launched in 2022. This topic was taken for study based on the felt need of the Program Manager.  Although designed as a descriptive study, by assessing coverage, timeliness, and equity against program expectations, our first of its kind analysis offers an evaluative perspective of the *Ni-kshay Mitra* initiative. The findings highlight specific implementation gaps such as limited coverage (half of expected supply), delayed initiation (median 41 days), and lack of equity prioritization, thereby providing actionable evidence for program strengthening. (somewhere after key findings in discussion para. ) |  |
| **3** | The title specifies coverage, time, who received the basket while the objectives are about characteristics of Nikshay Mitras, models, coverage, time interval, facilitating factors and barriers of implementation. | Coverage, Delivery models, and Implementation Challenges of the Ni-kshay Mitra Food Basket Initiative for People with TB: a mixed method study from Puducherry, India” | Page no 1. Line no:3-5 |
| **4** | Enlisted objectives appear to be vague and vary significantly from each other. | **We have reworded our objectives for better coherence.**  **Revised objective being to**  i) describe the characteristics of Ni-kshay Mitras and document the various models of food basket distribution;  ii) determine the coverage of food basket distribution during the first 6 months of treatment and time interval in the initiation of first food basket from diagnosis, and  iii) understand the facilitators and barriers to implementation from the perspective of health care providers, *Ni-kshay Mitras* and adults with TB | Page no 5: Line no 89-94 |
| **5** | There is no coherence and findings may not contribute to the improvement of ongoing programme. | Thank you for this observation. We have revised the discussion and recommendations section to explicitly link our findings with the ongoing *Ni-kshay Mitra* initiative. In addition, the barriers and facilitators identified have been translated into actionable, program-integrated recommendations.  We believe such recommendations strengthens the coherence of the findings and highlights their direct relevance for program improvement. Also, we have began implementation research on how to address inequity in the distribution of food baskets as a part of the year 2 course. As the study is ongoing, the results of it will be available following its publication next year. | Page no:22, Line no: 483-485 |
| **6** | Justify how the data on the characteristics of Nikshay Mitra contribute to this study. | Thank you for the comment. This information served as a basis for purposively selecting the Ni-Kshay Mitra’s for in-depth interviews. It has been added in the write up. | Page no 12, Line no: 247-249 |
| **7** | Line 487 - Explain how Ni-Kshay Mitras were linked to the consented adults with TB within their geographical setting – is a challenge | Thank you for the comment. As majority of the Nikshay Mitras in Puducherry were individual mitras, they generally prefer distribution of food baskets to their nearest health facility. This is a challenge in areas where there are no *Ni-kshay mitras*. This has been mentioned in the discussion section. | Page no 20, Line no: 440-441 |
| **8** | Line 120 – change in weight or change in weight gain. Add justification in the write-up why to discuss about weight gain, when there is no data available for the addressing the question. | Thank you for the comment. The following write up has been added: Despite the availability of logistics and the provision for documentation of weight in the treatment card, documentation gap exists. The reason being absenteeism by PwTB during follow-up visits and the receival of food baskets by their family members. Counselling PwTB on the importance of weight measurement during follow up visits, along with real-time documentation by health care providers, is crucial for bridging the gap. | Page no 20, Line no: 427-432 |
| **9** | Rather than describing Quan and Qual under each heading, it would have been better to write it according to the sequence followed in conducting the study | Thank you for the comment and we value it. We have discussed it among the co-authors. We would like to present our findings in a reader friendly manner to the target audience such as policy makers and researchers. Hence, we would like to retain the current format of the manuscript in order to highlight the integration of two designs in a simple manner. |  |
| 10 | Avoid repetition of information in the manuscript. Example, specifying dates in multiple sentences. Also, there is a mismatch of dates specified in Figure 1. | Thank you for the comment. Previous mention of duration in the main text has been removed.  We would like to clarify that there is no mismatch of date in the Figure 1, as the objective 2 in the figure corresponds to the time frame in which the secondary data was obtained.  While the time frame in the title of Figure 1 corresponds to the study period following ethics approval. |  |
| 11 | Table 2 – as the Kurt Lewin framework has been used, a better representation of data will be appreciated | Thank you for the comment. Our study comprises of vast qualitative data from various stakeholders. We would like to propose solutions in par with the challenges and available resources at our study setting. Therefore, we opted for representation of the data as a table in order to facilitate easy understanding. |  |
| 12 | Line 96 – 105: The description about the pragmatic paradigm, appears inappropriate along with study designs. The suggestion is to discuss it as a strength of the study. Avoid the term retrospective cohort. | Thank you for the comment. The point on pragmatic paradigm has been moved to the strengths section. | Page no 21: Line no: 457-459 |
| 13 | The entire section looks poorly structured. Instead of detailed description, I encourage using tables, graphs, diagram or models. Eg: simple table or bar chart showing distribution of timing (e.g., <2 weeks, 2–4 weeks, >4 weeks) | Thank you for the comment. In this manuscript, we have included three tables and three figures. Tables have been provided towards the end of the manuscript and figures as separate .tif file (flowcharts and models).  Further, the description in the results section is only a brief summary and it includes information that is not provided within the table/ figure. |  |
| 14 | Qualitative analysis – for many questions, only data has been provided in the form of quotes. There is no description of results. Eg: What are the results regarding the quality of the food baskets | Thank you for the comment. Necessary changes have been made in the results section. | Page no 13: Line no: 277-278  Page no 14: Line no: 305-306  Page no 14: Line no: 314-316. |
| 15 | Line 391 – why there is an equity gap when – ‘observed that all adults with TB were distributed food baskets irrespective of their nutritional and socio-economic status since 2022.’ Line 407 and ‘These indicators were similar across different socio-economic and nutritional statuses.’ Line 473 | Thank you for the comment. As per our operational definition, equity was assumed if the following indicators were better among adults with very severe undernutrition and below poverty line.   - Monthly food baskets received per adult with TB as median (IQR) - Interval between treatment initiation and receipt of first basket as median (IQR).   However, there was no difference in these two vulnerable groups-based n findings provided in Table 3. Therefore, we concluded the statement as “all adults with TB were distributed food baskets irrespective of their nutritional and socio-economic status since 2022”. |  |
| 16 | Line 403 - Avoid including multiple concept texts in a single sentence. Eg: what is the relevance of the time of receiving basket when the sentence is on the equity of distribution. It hinders the flow of reading and difficult to comprehend. | Thank you for the comment. As per our operational definition, equity was assumed if the following indicators were better among adults with very severe undernutrition and below poverty line.   - Monthly food baskets received per adult with TB as median (IQR) - Interval between treatment initiation and receipt of first basket as median (IQR).   Hence, we have added in the paragraph on equity of food baskets. |  |
| 17 | Line 411 and 437 – Monitoring weight gain during treatment is mandatory. Failure to do so, shows the implementation gap. It should be discussed and cannot be included as a recommendation | Thank you for the comment. Has been removed from the recommendation sections and explained briefly in the discussion section. | Page no 20, Line no: 440-441 |
| 18. | Implication:  Implication of delay should be discussed – suggestion is to include the effect of undernutrition on TB outcome etc. | Thank you for the comment. The effect of early nutrition support on treatment outcome has been added with references. | Page no 19, Line no: 420-422 |
| 19. | Explicitly acknowledge the limitations – secondary data, incomplete data, risk of misclassification of socio-economic status etc. | Thank you for the comment. Necessary changes have been made in the limitations section. | Page no 21, Line no: 463-465 |
| 20. | Recommendation:  Make it more actionable by incorporating it to the existing programme. Eg: procuring the baskets one month prior etc. | Thank you for the comment. It has been added under recommendations. | Page no 22, Line no: 483-485 |
| 21. | Conclusion:  Avoid restating findings – add programmatic changes which are required to address the issue. | Thank you for the comment. We have highlighted the programmatic recommendations under conclusion section. | Page no 22-23, Line no: 499-503 |
