# Supplementary material for: Coverage, delivery models, and implementation challenges of the community driven nutritional supplementation initiative for people with TB: A mixed methods study from Puducherry, India
Source: PLOS Glob Public Health. 2025 Dec 23;5(12):e0005477. doi: 10.1371/journal.pgph.0005477 (PMC12725536; doi:10.1371/journal.pgph.0005477)
Supplement: Attachment — Submitted filename: Response to the journal.docx [file pgph.0005477.s008.docx]

| **Sl No** | **Journal comments** | **Reply to the journal comments** | **Reference** |
| --- | --- | --- | --- |
| 1 | If a manuscript's minimal data set cannot be made publicly available, PLOS requires that authors provide a Data Availability statement with specific information to facilitate data access requests.  For data that is subject to restrictions, we ask that authors include the following information in their statement: -The reason for the restriction (i.e., confidentiality of patients, etc.) -The name of the restricting institution -A non-author, institutional point of contact (preferably email) for a data access committee, ethics committee, or other institutional body that other researchers would require to request access to your data.  Note that it is not acceptable for an author to be the sole named individual responsible for ensuring data access. | Following discussion with the co-investigators we have decided that:  The minimal data set underlying the findings of this study contains sensitive and identifiable information about persons with tuberculosis and cannot be shared publicly to protect participant confidentiality. Data access is therefore restricted by the State Tuberculosis Office, Government of Puducherry.  Researchers who meet the criteria for access to confidential data may submit a reasonable request in writing to the State Tuberculosis Officer (Email: [stopd@rntcp.org](mailto:stopd@rntcp.org)) through the Department of Community Medicine, Sri Manakula Vinayagar Medical College and Hospital, Puducherry (Email: [info.commedicine@smvmch.ac.in](mailto:info.commedicine@smvmch.ac.in)).  The corresponding author will facilitate communication but will not be responsible for approving access. | This has been mentioned in the data availability statement in the submission portal. |
| 2 & 3 | Please amend your detailed online Financial Disclosure statement. This is published with the article. It must therefore be completed in full sentences and contain the exact wording you wish to be published.   Please ensure that the funders and grant numbers match between the Financial Disclosure field and the Funding Information tab in your submission form. Note that the funders must be provided in the same order in both places as well. | Thank you for the comment. The Financial disclosure statement has been added as follows in the manuscript.  The modular training under this SORT IT course was funded by the ICMR-National Institute of Epidemiology (ICMR-NIE), Chennai, India. ICMR-NIE did not provide any specific funding for the operational/ implementation research (that resulted in this manuscript) conducted through this SORT IT course. The research was conducted in routine operational settings utilizing existing health system resources and workforce. Open access publication fee was supported by ICMR-NIE Chennai, India.  The same has been ensured in the Funding Information tab as well. | Refer to the manuscript in Page no 24. Line no: 527-532. |
| 4 | Can you please upload copies of your Supporting Information files that does not contain any tracked changes, comments, or highlighting. | Thank you for the comment. Has been uploaded. |  |
